# Excessive Smartphone Use and Self-Esteem Among Adults With Internet Gaming Disorder: Quantitative Survey Study

**DOI:** 10.2196/18505

**Published:** 2020-09-29

**Authors:** Hyunmin Kim, In Young Choi, Dai-Jin Kim

**Affiliations:** 1 Department of Medical Informatics College of Medicine The Catholic University of Korea, Seoul St. Mary's Hospital Seoul Republic of Korea; 2 Division of Health Systems Management and Policy School of Public Health The University of Memphis Memphis, TN United States; 3 Department of Psychiatry College of Medicine The Catholic University of Korea, Seoul St. Mary’s Hospital Seoul Republic of Korea

**Keywords:** excessive smartphone use, internet gaming disorder, smartphone overuse, self-esteem, mental health, gender difference, Korean smartphone addiction proneness scale, smartphone, gaming, young adult, adult, gender

## Abstract

**Background:**

Smartphone overuse can harm individual health and well-being. Although several studies have explored the relationship between problematic or excessive smartphone use and mental health, much less is known about effects on self-esteem, which is essential in having a healthy life, among adults with mental health disorders, including internet gaming disorder. Furthermore, given that smartphone usage differs by gender, little is known about gender differences in the relationship between smartphone overuse and self-esteem.

**Objective:**

The objective of this study was to assess self-esteem among individuals with mental health disorders and explore the relationship with excessive smartphone use.

**Methods:**

Participants were selected based on their responses to the internet gaming disorder assessment, which includes 9 items developed based on Diagnostic and Statistical Manual of Mental Disorders (Fifth Edition) criteria, from among a Korean cohort of smartphone users aged 20-40 years, resulting in a sample of 189 participants (men:120, women: 69). The Rosenberg self-esteem scale and the Korean smartphone addiction proneness scale were utilized to assess the outcome self-esteem with excessive smartphone use as the primary independent variable. Guided by the Bowlby attachment theory and prior studies, we selected several covariates. Generalized linear regression analyses, as well as subgroup analyses by gender, were performed.

**Results:**

Among adults with internet gaming disorder, the average Korean smartphone addiction proneness scale score was significantly higher in women than that in men (41.30 vs. 37.94; *P*=.001), and excessive smartphone use was significantly more prevalent in women than it was in men (30.43% vs. 20.83%; *P*=.02). Our findings from the generalized linear regression analyses indicated that an increase in Korean smartphone addiction proneness scale score had a negative relationship with self-esteem among those with internet gaming disorder (β=–0.18, *P*=.001). Furthermore, our interaction models showed that, among those with internet gaming disorder, more men than women had lower self-esteem associated with an increase in Korean smartphone addiction proneness scale score and a high degree of smartphone overuse (β=–0.19, *P*=.004; β=–3.73, *P*<.001).

**Conclusions:**

Excessive smartphone use was found to be adversely associated with self-esteem among young and middle-aged adults with internet gaming disorder; notably, more men than women were negatively influenced (regarding self-esteem) by smartphone overuse. Based on our findings, more efforts should be made to reduce excessive or problematic smartphone use by considering developing public health interventions or policy, particularly among those with mental health disorders such as internet gaming disorder.

## Introduction

In the era of the internet and with fast-growing mobile technology availability, smartphones have become an essential part of people’s daily lives because of their mobility and various functions. Without bringing a laptop everywhere, people can easily send emails, have a video conference, share files and photos, and have access to entertainment (eg, playing games, etc) by using smartphones. According to a 2015 Pew Research Center report [1], almost two-thirds of the adults in the United States own a smartphone that 46%, among them, mentioned they “couldn’t live without [1].” Among other developed countries, South Korea is known for having the highest smartphone penetration and use; it was estimated that approximately 95% of South Koreans used a smartphone in 2018, which was the top rate among the 27 nations that responded to the survey [2]. However, with substantial growth in the smartphone market, a related issue—excessive or problematic smartphone use—has arisen. Evidence indicates that many people use smartphones to affect their daily lives negatively [3,4]. Constant checking of the phones and using apps all day long could exemplify excessive smartphone use, which can also result in social issues [5].

Growing evidence suggests that excessive or problematic smartphone use can adversely affect individual physical health (eg, headaches, neck, and wrist pain; obscured vision) [6,7] and mental health (eg, depression, anxiety, etc) [8-11] as well as psychological attributes such as self-esteem and self-control [10,12,13]. For instance, a systematic review by Elhai and colleagues [10] explored the relationship of psychopathology with problematic smartphone use and identified anxiety, depression, and low self-esteem as psychopathological correlates. Notably, self-esteem, defined as “the degree that a person likes, values, and approves himself or herself [14],” is essential in having a healthy life and for psychological well-being [15]. Self-esteem provides positive outcomes and benefits in life, for example, being confident in one's own abilities can help decision making and in being more resilient in coping with stress and difficulties [15]. However, evidence shows that individuals with mental health disorders such as depression, anxiety, eating disorders, and personality disorders are more likely to have low self-esteem or self-esteem deficits [16].

To explore the relationship of self-esteem with extent of excessive smartphone use, the Bowlby attachment theory [17-19] may be applied as a theoretical base. According to this theory [17-19], there are 3 attachment styles: secure, avoidant, and anxious-ambivalent. The first type, “secure attachment,” can be characterized by a higher sense of self-worth, considering others trustworthy and reliable, and controlling consequences in their lives. The second type, “avoidant,” is characterized as have a lower sense of self-worth and viewing others as less kind, while the third type, “anxious-ambivalent,” is characterized as feeling insecure about others’ responses and having a strong desire for closeness to others. Therefore, people with avoidant and anxious-ambivalent attachment styles are more likely to have negative beliefs about themselves but relatively positive views about others, which could make them more reliant on others by seeking their authorization and reassurance [20]. In this context, a mobile phone or smartphone could serve as a means for them to receive the approval and reassurance from people close to them. Evidence indicates that low self-esteem related to insecure attachment could play a role in the excessive use of mobile phones or smartphones [21,22].

Although prior research has explored relationships between excessive or problematic smartphone use and mental health or psychological attributes among general populations, much less is known about effects on self-esteem among people with mental health disorders such as internet gaming disorder [23]. Those who are addicted to online gaming tend to have specific characteristics, such as obsession, withdrawal, and loss of interest in doing other things (ie, job-related or educational activities) [24,25]. Given that low self-esteem or self-esteem deficits may be more likely to occur among individuals with mental health disorders, it is meaningful to assess self-esteem related to problematic smartphone use among these groups. Additionally, given that smartphone usage differs by gender [26,27], it is also worthwhile to examine whether there exist gender differences in the relationship between smartphone overuse and self-esteem, about which little is known. We believe that this study has contributed to filling gaps in the literature and could help in developing public health interventions and policy to reduce excessive smartphone use among adults with mental health disorders, particularly those with internet gaming disorder.

## Methods

### Data and Study Participants

This study included individuals recruited from various parts of South Korea (Metropolitan area, Chungcheong-do, Gyeongsang-do, Jeolla-do, and Gangwon-do/Jeju-do) from October 2018 to February 2019 through the smartphone overdependence management system. The smartphone overdependence management system is a mobile app–based system, developed in a prior study [28], to identify and examine factors associated with smartphone overdependence that can help prevent and monitor smartphone overreliance. Thus, the smartphone overdependence management system provided us with information about excessive smartphone use. In the initial research, the inclusion criteria were (1) aged 20-40 years; (2) Android -based smartphone users; and (3) smartphone usage of at least one hour or more per day. To retain participants for 4 weeks, the project utilized a specialized company to recruit and manage participants, who were randomly selected. All participants received a small reward for their participation through the company. Since this study focused on individuals with internet gaming disorder, we utilized a questionnaire for internet gaming disorder assessment that consisted of 9 items (preoccupation, tolerance, withdrawal, persistence, escape, problems, deception, displacement, and conflict) [29] that were developed based on Diagnostic and Statistical Manual of Mental Disorders Fifth Edition (DSM-5) criteria [24]. Each of the 9 items was coded as binary (1=yes, 0=no) and summed for a total score. Using the conventional cutoff point for internet gaming disorder, we selected those with a total of 5 or above as an indication of internet gaming disorder [24] (ie, 0-4 for normal; 5-9 for internet gaming disorder), which resulted in a sample of 189 participants with internet gaming disorder (men: 120/189, 63.5%; women: 69/189, 36.5%). A survey questionnaire (written in Korean) was utilized to obtain individual-level information (social and demographic characteristics including age, marital status, education, and income; family-related characteristics such as receipt of emotional support from family and relationship with family; and adverse conditions including perceived abuse experience and experience of being bullied). The study was approved by the institutional review board of the Catholic University of Korea, Seoul St. Mary’s Hospital (approval no. MC16QISI0146). All participants signed an informed consent form before the study in accordance with the Declaration of Helsinki.

### Measures

#### Dependent Variable

The Korean version of the Rosenberg self-esteem scale [30] was used to evaluate individual *self-esteem*. This scale consisted of 10 items with a 4-point Likert scale (4=strongly agree, 3=somewhat agree, 2=somewhat disagree, and 1=strongly disagree). The example questionnaire statements for evaluating self-esteem included “I take a positive attitude toward myself,” “I feel that I have many good qualities [31].” In a prior study [32], the Rosenberg self-esteem scale was found to have relatively high reliability and consistency (Cronbach α=.72). The self-esteem scale ranged between 0 and 40, in which a higher total score indicated a higher level of self-esteem.

#### Independent Variables

The Korean Smartphone Addiction Proneness Scale (K-SAPS), developed by the National Information Agency of Korea in 2011, was used to assess *excessive smartphone use*. The K-SAPS, which additionally includes items with unique smartphone features in the Internet Addiction Proneness Scale for Youth, was found to be highly reliable (Cronbach α=.88) in prior research [33]. The K-SAPS comprised 15 items in total with a 4-point Likert scale (4=strongly agree, 3=somewhat agree, 2=somewhat disagree, and 1=strongly disagree) and is appropriate for screening addictive smartphone use [33]. The K-SAPS consisted of 4 subdomains: disturbance of adaptive functions (5 items), virtual life orientation (2 items), withdrawal (4 items), and tolerance (4 items). Disturbance of adaptive functions was assessed, for example, with items such as “People frequently comment on my excessive smartphone use” and “Family or friends complain that I use my smartphone too much.” Withdrawal, for example, was measured with items such as “It would be unpleasant if I am not allowed to use a smartphone” and “I become anxious and restless when I do not have a smartphone with me.”

We included the K-SAPS as the primary independent variable in the model and also examined it as a categorical variable to assess the degree of excessive smartphone use (high, moderate, and low) based on prior research [34]. A high degree of excessive smartphone use was defined as K-SAPS≥44 or by subdomain scores ≥15 for adaptive function or ≥13 for both tolerance and withdrawal. A moderate degree of excessive smartphone use was defined as 40≤K-SAPS≤43 or any subdomain score≥14. A low degree of excessive smartphone use was defined as any not meeting these criteria.

#### Covariates

Guided by the Bowlby attachment theory [17-19] and empirical studies examining factors related to self-esteem [12,35,36], we selected several covariates. They included social and demographic factors (age, gender, marital status, education, and income), family-related factors (emotional support from family, relationship with the family), as well as adverse conditions (perceived abuse experience, experience of being bullied). *Age* was used as a continuous variable in this study. *Marital status*, indicating whether or not an individual was currently married, was grouped into 3 categories: never been married, married, separated/widowed. *Education*, indicating the highest level of educational attainment an individual achieved, was included as a categorical variable (less than high school, high school graduate, college graduate or above). *Income* (*X* Korean won or KRW; an approximate exchange rate of 2,000,000 KRW=US $1713 was applicable at the time of publication), was measured by the average monthly household income before tax categorized into *X*<2,000,000, 2,000,000≤*X*<4,000,000, 4,000,000≤*X*<6,000,000, and *X*≥6,000,000. The variable *emotional support from family* (not at all, some, a lot) was included. It was constructed based on the survey question, “How much do you think you receive emotional support from your family?” The variable *relationship with the family* (less satisfied/dissatisfied, satisfied, very/completely satisfied) was also included based on the survey question “How satisfied are you with the relationship with your family?” Additionally, we included *perceived abuse experience* and *experience of being bullied* as binary variables, assessed based on the survey questions “Have you ever felt that you received abuse from your family or someone who provided care when you were a child?” and “Have you ever experienced being bullied at school?” respectively.

### Statistical Analysis

The dependent variable in this study was self-esteem, measured with the Rosenberg self-esteem scale. The independent variables were K-SAPS score and degree of excessive smartphone use (high, moderate, or low). Using descriptive statistics, we first examined the characteristics of the Korean cohort of young and middle-aged adults with internet gaming disorder and differences by gender. Specifically, we compared the extent to which men and women were different concerning individual characteristics using two-tailed independent *t* tests for continuous variables and Rao-Scott chi-square tests for dichotomous variables. The threshold of .05 was used for assessing the statistical significance of those tests.

Furthermore, we utilized a generalized linear regression model to examine the association between K-SAPS and self-esteem and between the degree of excessive smartphone use and self-esteem, among adults with internet gaming disorder. The Rosenberg self-esteem scale was not normally distributed (Shapiro-Wilk normality test: *P*<.001). We conducted generalized linear regression analyses by employing 4 distinct models: Model 1 included K-SAPS score as the primary independent variable without any interaction terms. Model 2 included the degree of excessive smartphone use as the primary independent variable without any interaction terms. Model 3 included the interaction between gender and K-SAPS score. Model 4 included the interaction between gender and degree of excessive smartphone use. Notably, the interaction models were constructed based on the results from the statistical tests comparing men and women. All statistical analyses were conducted using SAS (version 9.4; SAS Institute) statistical software.

## Results

### Characteristics of Study Participants

Table 1 presents the characteristics of study participants by gender. The average K-SAPS (41.30 vs. 37.94; *P=*.001) and frequency of a high degree of excessive smartphone use (30.43% vs. 20.83%; *P=*.02) were significantly higher for women than those for men. Except for the K-SAPS and degree of excessive smartphone use, however, no statistically significant differences by gender were shown concerning individual characteristics. The mean Rosenberg self-esteem scale (24.17 vs. 24.07; *P=*.88) was slightly higher for men than that for women. The average age (34.45 years vs. 34.30 years; *P=*.90) for men was higher than that for women. Being married (49.28% vs. 41.67%; *P=*.28), and with higher incomes (4,000,000 earned and above) (50.72% vs. 31.67%; *P=*.28) were more common in women than they were in men. College graduate or more education (81.67% vs. 73.91%; *P=*.29) was more prevalent in men than it was in women. Receipt of emotional support from family (some and a lot) (88.41% vs. 85.83%; *P=*.84) was more common in women than it was in men, while being satisfied with the family relationship (89.17% vs. 88.41%; *P=*.74) was more prevalent in men than it was in women. Perceived abuse experiences (33.33% vs. 23.33%; *P=*.13) and incidents of being bullied at school (43.48% vs. 31.67%; *P=*.10) were more common in women than they were in men.

**Table 1:** Characteristics of the study participants.

Characteristic		All (N=189)	Men (n=120)	Women (n=69)	*P* value^a^
K-SAPS^b^, mean (SD)		39.16 (6.91)	37.94 (7.06)	41.30 (6.13)	.001
**Degree of excessive smartphone use, n (%)**					.02
	High (K-SAPS≥44)	46 (24.3)	25 (20.8)	21 (30.4)	
	Moderate (40≤K-SAPS≤43)	52 (27.5)	28 (23.3)	24 (34.8)	
	Low (K-SAPS≤39)	91 (48.2)	67 (55.8)	24 (34.8)	
Social and demographic					
Age (in years), mean (SD)		34.39 (7.9)	34.45 (8.11)	34.30 (7.61)	.90
**Marital status, n (%)**					.28
	Never been married	102 (54.0)	69 (57.5)	33 (47.8)	
	Married	84 (44.4)	50 (41.7)	34 (49.3)	
	Separated/widowed	3 (1.6)	1 (0.8)	2 (2.9)	
**Education, n (%)**					.29
	Less than high school	1 (0.5)	1 (0.8)	0 (0.0)	
	High school graduate	39 (20.6)	21 (17.5)	18 (26.1)	
	College graduate or above	149 (78.8)	98 (81.7)	51 (73.9)	
**Income (X KRW^c^ /month), n (%)**					.28
	X<2,000,000	33 (17.5)	23 (19.2)	10 (14.5)	
	2,000,000≤X<4,000,000	77 (40.7)	53 (44.2)	24 (34.8)	
	4,000,000≤X<6,000,000	45 (23.8)	24 (20.0)	21 (30.4)	
	X≥6,000,000	34 (18.0)	20 (16.7)	14 (20.3)	
**Emotional support from family, n (%)**					.84
	Not at all	25 (13.2)	17 (14.2)	8 (11.6)	
	Some	123 (65.1)	78 (65.0)	45 (65.2)	
	A lot	41 (21.7)	25 (20.8)	16 (23.2)	
**Relationship with the family, n (%)**					.74
	Less satisfied/dissatisfied	21 (11.1)	13 (10.8)	8 (11.6)	
	Satisfied	68 (36.0)	41 (34.2)	27 (39.1)	
	Very/completely satisfied	100 (52.9)	66 (55.0)	34 (49.3)	
**Perceived abuse experience, n (%)**					.13
	Yes	51 (27.0)	28 (23.3)	23 (33.3)	
	No	138 (73.0)	92 (76.7)	46 (66.7)	
**Experience of being bullied, n (%)**					.10
	Yes	68 (36.0)	38 (31.7)	30 (43.5)	
	No	121 (64.0)	82 (68.3)	39 (56.5)	
Rosenberg Self-Esteem Scale, mean (SD)		24.13 (4.82)	24.17 (4.79)	24.07 (4.92)	.88

^a^Significance assessment of the Rao-Scott chi-square test for categorical variables and *t* test for continuous variables. The significance level of .05 was incorporated for the assessment.

^b^K-SAPS: Korean Smartphone Addiction Proneness Scale.

^c^KRW: Korean won (an approximate exchange rate of 2,000,000 KRW=US $1713 was applicable at the time of publication).

### Association Between Excessive Smartphone Use and Self-Esteem Among Adults With Internet Gaming Disorder

Table 2 shows the results of our 4 generalized linear regression models for examining the estimated effects of the K-SAPS and the degree of excessive smartphone use on self-esteem among adults with internet gaming disorder. Model 1 showed an increase in the K-SAPS had a negative relationship to self-esteem (β=–0.18, *P=*.001) among adults with internet gaming disorder. Model 2 showed that compared with those with a low degree of excessive smartphone use, individuals with internet gaming disorder with a high degree of excessive smartphone use had lower self-esteem (β=–3.42, *P<*.001). Furthermore, all models showed that compared with adults with internet gaming disorder who were very/completely satisfied with their family relationship, those who were less satisfied/dissatisfied with their family relationship had lower self-esteem (model 1: β=–5.21, *P<*.001; model 2: β=–5.55, *P<*.001; model 3: β=–5.43, *P<*.001; model 4: β=–5.63, *P<*.001). Meanwhile, interactions showed that among adults with internet gaming disorder, men had lower self-esteem associated with an increase in the K-SAPS than women (β=–0.19, *P=*.004). Additionally, among adults with internet gaming disorder, men with a high degree of excessive smartphone use had lower self-esteem than women (β=–3.73, *P<*.001).

**Table 2:** Generalized linear models of self-esteem concerning smartphone overuse among adults with internet gaming disorder.

Variables		Self-esteem^a^							
		Model 1		Model 2		Model 3		Model 4	
		B^b^	*P* value	B	*P* value	B	*P* value	B	*P* value
K-SAPS^c^		–0.18	.001	N/A^d^	N/A	N/A	N/A	N/A	N/A
**Degree of excessive smartphone use (reference: low)^e^**									
	Moderate	N/A	N/A	–0.13	.87	N/A	N/A	N/A	N/A
	High	N/A	N/A	–3.42	<.001	N/A	N/A	N/A	N/A
Social and demographic									
Age (in years)		0.03	.52	0.03	.48	0.04	.40	0.04	.48
Gender (reference: men)		–0.66	.66	–0.51	.47	N/A	N/A	N/A	N/A
**Marital status (reference: separated/widowed)**									
	Never been married	-6.94	.05	–5.78	.11	–6.78	.06	–5.76	.11
	Married	-6.81	.05	–5.96	.08	–6.76	.05	–5.93	.09
**Education (reference: college graduate or above)**									
	Less than high school	–4.90	.13	–4.53	.15	–4.69	.14	–4.52	.15
	High school graduate	–0.94	.94	–1.18	.15	–0.58	.48	–1.14	.17
**Income (X KRW** ^f^ **/month) (reference: ≥6,000,000)**									
	X<2,000,000	1.49	.24	1.97	.12	1.46	.24	1.99	.12
	2,000,000 ≤X<4,000,000	1.09	.28	1.49	.14	1.06	.29	1.56	.13
	4,000,000 ≤X<6,000,000	2.37	.02	2.87	.01	2.28	.03	2.90	.01
Family-related									
**Emotional support from family (reference: a lot)**									
	Not at all	–0.37	.76	–0.55	.65	–0.59	.64	–0.55	.65
	Some	–0.45	.61	–0.65	.45	–0.47	.60	–0.63	.47
**Relationship with the family (reference: very/completely satisfied)**									
	Less satisfied/dissatisfied	–5.21	<.001	–5.55	<.001	–5.43	<.001	–5.63	<.001
	Satisfied	–1.47	.06	–1.50	.05	–1.58	.04	–1.50	.05
**Adverse conditions**									
	Perceived abuse experience	–0.93	.27	–1.05	.20	–1.04	.21	–1.09	.18
	Experience of being bullied	–0.11	.88	–0.02	.97	–0.15	.84	0.03	.96
**Interactions**									
	Gender×K-SAPS^g^	N/A	N/A	N/A	N/A	–0.19	.004	N/A	N/A
	Gender×degree of excessive smartphone use: high	N/A	N/A	N/A	N/A	N/A	N/A	–3.73	<.001

^a^The Korean version of the Rosenberg self-esteem scale was used for the outcome assessment.

^b^B is the unstandardized coefficient.

^c^K-SAPS: Korean Smartphone Addiction Proneness Scale.

^d^N/A: not applicable.

^e^Degree of excessive smartphone use was assessed by the K-SAPS: high (K-SAPS≥44), moderate (40≤K-SAPS≤43), and low (K-SAPS≤39).

^f^KRW: Korean won (an approximate exchange rate of 2,000,000 KRW=US $1713 was applicable at the time of publication).

^g^Grand mean centering and scaling (dividing by SD) was applied to the continuous K-SAPS variable.

## Discussion

### Principal Findings and Implications for Public Health Interventions and Policy

To the extent of our knowledge, this study is the first, based on a Korean cohort of young and middle-aged adults with internet gaming disorder, to examine the association between excessive smartphone use and self-esteem and detect gender differences. Although several studies have investigated the relationship between problematic or excessive smartphone use and mental health [8-10,21] and psychological attributes [12,13], much less is known about the effects on self-esteem among those with mental health disorders, including internet gaming disorder. Smartphone overuse could play a role as a stimulus for gaming (by using mobile apps), which can adversely affect smartphone users’ health and well-being. There may be reason for concern for those with internet gaming disorder because they may be more prone to utilizing smartphones for gaming, with features such as accessibility and mobility, which could increase online gaming dependence. In this study, we found that excessive smartphone use was adversely associated with self-esteem in adults with internet gaming disorder. Given a substantial increase in smartphone usage, developing public health interventions or programs to reduce smartphone overuse should be considered, particularly for those with mental health disorders such as internet gaming disorder.

Another finding of this study was that men were found to be more negatively influenced from excessive smartphone use concerning self-esteem than women were; notably, men with a high degree of smartphone overuse were more likely to have low self-esteem compared with women with a high degree of smartphone overuse. This finding may be explained with the circumstance that men may use their smartphones, specifically for gaming, or their gaming behavior may be carried out via their smartphone. In contrast, women may game on other internet-connected devices and use their smartphones for different functions, including social networking or information searching [27]. In other words, women may socialize more by using their smartphones or mobile phones than men do, which could more positively affect self-esteem in women than that in men. A meta-analysis by Harris and Orth [37] examined the relationship between social relationships and self-esteem with 52 longitudinal studies published between 1993 and 2016. They found a reciprocal link between self-esteem and social relationships. Specifically, positive social relationships, acceptance, and support seemed to affect self-esteem positively among individuals across all levels of development [37].

Interestingly, among adults with internet gaming disorder, those who perceived less satisfaction or dissatisfaction about their relationships with their family seemed to have lower self-esteem than those who were very/completely satisfied with their relationships with their family. Indeed, prior research identified family-related factors as predictors of self-esteem among young individuals, including family functioning and environment [38], parent-child relationship [39], and parental attitudes toward their children (perceived parental rejection and criticism and parental fairness) [40]. Furthermore, evidence suggests that parental influences on self-esteem may vary by factors such as children’s age, development status, and gender. For instance, a longitudinal study [41] of 282 adolescents found that family influences (ie, perceptions about family communication) had a significant impact on self-esteem among young adolescents at Time 1; however, it appeared to not affect self-esteem among young adolescents at Time 2. This finding suggests that the effects of family factors on self-esteem may be higher among younger rather than older individuals [41]. In terms of gender in the relationship between parental influences and self-esteem, prior studies [42-44] reported varying results. For example, several reviews mentioned that family functioning and family relationships might be more influential on boys’ self-esteem than on girls' self-esteem [42,43]. However, other research conducted with a sample of 230 college students found that while parental authoritativeness and authoritarianism were shown to be predictive of self-esteem, female students were more likely than male students to be affected by their parents’ attitudes [44].

### Limitations and Strengths

This study had some limitations. First, this study mainly examined the estimated effects of excessive smartphone use on self-esteem, and gender differences, among young and middle-aged adults with internet gaming disorder. However, evidence indicates a bidirectional relationship between addictive behaviors and psychological factors [12]. Second, due to the cross-sectional nature of the study, we may not infer a causal relationship between excessive smartphone use and self-esteem. Next, despite using measures for self-esteem and excessive smartphone use, which were found to be valid and reliable in prior research [32,33], the 4-point Likert scale (strongly agree, somewhat agree, somewhat disagree, strongly disagree) may not have been able to capture correct answers to the survey questions completely. For instance, some respondents might have intended to answer somewhere between strongly agree and somewhat agree or between strongly disagree and somewhat disagree; therefore, there may have been response bias by being limited to a scale with only 4 points [45].

Despite the limitations, there were several strengths. First, this study was conducted based on unique data, including a wide range of information about smartphone usage, internet gaming disorder, self-esteem, family-related factors, adverse experiences among young and middle-aged adults in South Korea. This allowed us to adjust for various related factors in associations between excessive smartphone use and self-esteem, which resulted in meaningful results, such as family-related factors. Second, this study, is the first (of which we are aware) to examine the relationship between excessive smartphone use and self-esteem among adults with internet gaming disorder, a population that was less explored in prior research. Finally, we further investigated gender differences in the association between smartphone overuse and self-esteem in this population, about which little is known.

### Conclusions

Although smartphones, with their advantageous features, have become an essential part of people’s daily lives, smartphone overuse could harm individual health and well-being. It is of particular concern for those more susceptible or vulnerable such as individuals with mental health disorders, including internet gaming disorder. Our finding of the negative association between excessive smartphone use and self-esteem among adults with internet gaming disorder suggests that more efforts or endeavors should be made by considering developing public health interventions or programs to reduce excessive or problematic smartphone use, particularly among those with mental health disorders such as internet gaming disorder. Furthermore, our finding of gender differences in the association between smartphone overuse and self-esteem could suggest merit in developing gender-specific interventions targeting men with internet gaming disorder.

## Abbreviations

KRW: Korean won
K-SAPS: Korean smartphone addiction proneness scale
