# Correction: Excessive Smartphone Use and Self-Esteem Among Adults With Internet Gaming Disorder: Quantitative Survey Study

**DOI:** 10.2196/24869

**Published:** 2020-11-03

**Authors:** Hyunmin Kim, In Young Choi, Dai-Jin Kim

**Affiliations:** 1 Department of Medical Informatics College of Medicine The Catholic University of Korea, Seoul St. Mary's Hospital Seoul Republic of Korea; 2 Division of Health Systems Management and Policy School of Public Health The University of Memphis Memphis, TN United States; 3 Department of Psychiatry College of Medicine The Catholic University of Korea, Seoul St. Mary’s Hospital Seoul Republic of Korea

In “Excessive Smartphone Use and Self-Esteem Among Adults With Internet Gaming Disorder: Quantitative Survey Study” (JMIR Mhealth Uhealth 2020;8(9):e18505), the authors noted one error in the Acknowledgments section.

In the originally published manuscript, the final sentence of the Acknowledgments section read “This work was supported by the National Research Foundation of Korea (NRF) (grant no. 2014M3C7A1062893).” The has been changed to: “This work was supported by the National Research Foundation of Korea (NRF) (grant nos. 2014M3C7A1062893 and NRF-2019R1A5A2027588).”

The correction will appear in the online version of the paper on the JMIR Publications website on November 3, 2020, together with the publication of this correction notice. Because this was made after submission to PubMed, PubMed Central, and other full-text repositories, the corrected article has also been resubmitted to those repositories.

